# Gastroparesis syndrome following thoracoscopic diaphragmatic hernia repair in a pediatric patient: a case report and literature review

**DOI:** 10.3389/fped.2026.1813576

**Published:** 2026-05-29

**Authors:** Zhang Tianyi, Hao Xiwei, Jiang Zhong, Ren Chuantao, Zhang Huanyu, Shang Cong, Dong Qian, Mi Jie

**Affiliations:** 1 Department of Pediatric Surgery, The Affiliated Hospital of Qingdao University, Qingdao, Shandong, China; 2 Department of Pediatric Surgery, Qilu Hospital of Shandong University Dezhou Hospital, Dezhou, China; 3 Shandong Provincial Key Laboratory of Digital Medicine and Computer-assisted Surgery, Qingdao, Shandong, China

**Keywords:** complication, congenital diaphragmatic hernia, gastroparesis syndrome, pediatric surgery, thoracoscopy

## Abstract

We present a rare case of postoperative gastroparesis syndrome (PGS) in a 10-year-old girl following thoracoscopic repair of congenital diaphragmatic hernia (CDH). The patient presented to our institution with a 20-day history of intermittent abdominal pain and vomiting. Thoracoabdominal computed tomography revealed a posterolateral congenital diaphragmatic hernia. Emergency thoracoscopic diaphragmatic repair was performed, after which the patient developed gastroparesis syndrome. The child was given continuous nasogastric suction, prokinetic therapy with domperidone, and acupressure massage at the Zusanli (ST36) acupoint. On the 38th day after the operation, the child was able to remove the nasal-gastric tube and resume normal diet. CDH is typically diagnosed and managed during infancy, with postoperative complications predominantly involving pulmonary hypertension and chronic lung disease. The occurrence of PGS following CDH repair is exceedingly rare. This report reviews the relevant literature to enhance clinicians’ awareness of this complication and improve patient outcomes.

## Introduction

Congenital diaphragmatic hernia represents a developmental anomaly characterized by incomplete formation of the diaphragm, permitting herniation of abdominal viscera into the thoracic cavity. While the majority of cases present during the neonatal period, late-presenting CDH in older children remains an uncommon but clinically significant entity. Thoracoscopic repair has emerged as the preferred surgical approach owing to its advantages of enhanced visualization, reduced postoperative pain, and accelerated recovery compared with conventional open thoracotomy (1, 2).

Postoperative gastroparesis syndrome constitutes a functional disorder of gastric motility occurring in the absence of mechanical obstruction, manifesting primarily as delayed gastric emptying (3, 4). Although PGS has been well-documented following pancreaticoduodenal and other major abdominal procedures, its occurrence after thoracoscopic diaphragmatic hernia repair in pediatric patients has not been previously reported in the literature.

Herein, we describe a case of PGS developing after thoracoscopic CDH repair in a 10-year-old girl and provide a comprehensive review of the pertinent literature to facilitate clinical recognition and management of this rare complication.

## Case presentation

A 10-year-and-9-month-old girl with no significant past medical history and no prior surgical interventions was admitted to our institution in October 2024 with a chief complaint of intermittent abdominal pain accompanied by vomiting for 20 days. The patient initially experienced paroxysmal periumbilical pain with four episodes of non-projectile vomiting. There was no associated chest tightness, dyspnea, or respiratory distress. Initial management at an outside facility with intravenous fluid resuscitation and antimicrobial therapy proved ineffective.

Subsequently, the vomiting became projectile in nature, with the emesis initially appearing black before transitioning to bilious green. The patient was transferred to our emergency department and admitted to the pediatric medicine service. Following surgical consultation, thoracoabdominal computed tomography was performed, and three-dimensional reconstruction utilizing the Hisense Computer-Assisted Surgery (CAS) system demonstrated a posterolateral congenital diaphragmatic hernia (Figures 1, 2).

**Figure 1:**
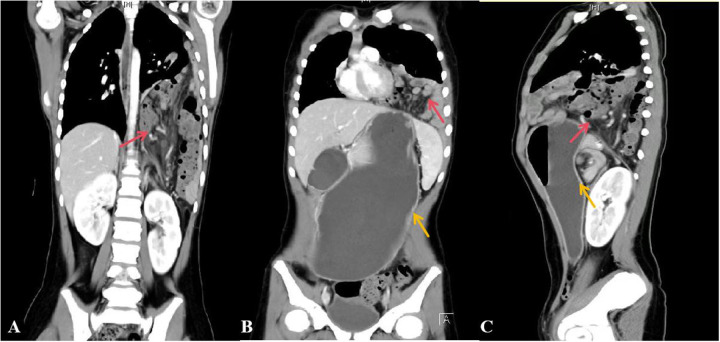
Preoperative CT imaging findings. The left thoracic cavity is occupied by extensive bowel loops, with marked gastric distension extending inferiorly to the pelvic cavity. **(A,B)** Coronal CT images; **(C)** sagittal CT image (red arrows indicate herniated bowel within the thoracic cavity; yellow arrows indicate the distended stomach).

**Figure 2:**
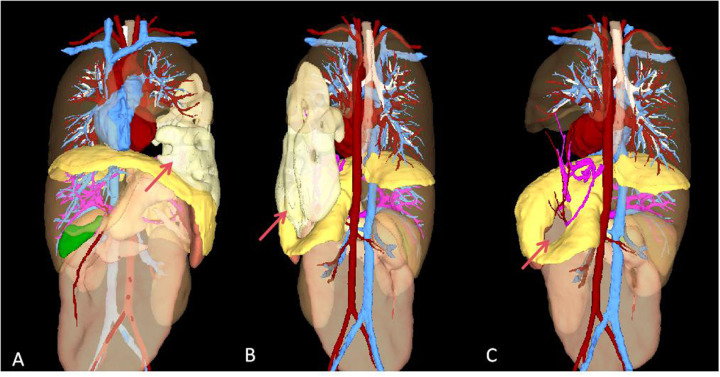
Preoperative Hisense CAS three-dimensional reconstruction. **(A)** Anterior view of CAS three-dimensional imaging (arrows indicate herniated bowel); **(B)** posterior view of CAS three-dimensional imaging (arrows indicate herniated bowel); **(C)** image following removal of bowel overlay (arrow indicates the hernia ring).

Physical examination revealed coarse breath sounds bilaterally with diminished breath sounds over the left lower lung field. No adventitious sounds were appreciated. The abdomen was scaphoid and soft with epigastric tenderness but without rebound tenderness. Bowel sounds were hypoactive. The admission diagnoses included incarcerated diaphragmatic hernia, electrolyte disturbance, and intestinal obstruction.

### Surgical intervention

Following nasogastric tube placement, emergency surgical intervention was undertaken. Intraoperative findings revealed extensive occupation of the left thoracic cavity by herniated bowel loops, which demonstrated adequate perfusion without significant adhesions to surrounding structures. Portions of the small intestine and ascending colon were identified herniating through a diaphragmatic defect measuring approximately 2 cm in its longest dimension. The operative diagnosis was confirmed as posterolateral congenital diaphragmatic hernia. After reduction of the herniated viscera into the abdominal cavity, partial diaphragmatic elevation was noted. Thoracoscopic diaphragmatic repair was completed without complication (Figure 3).

**Figure 3:**
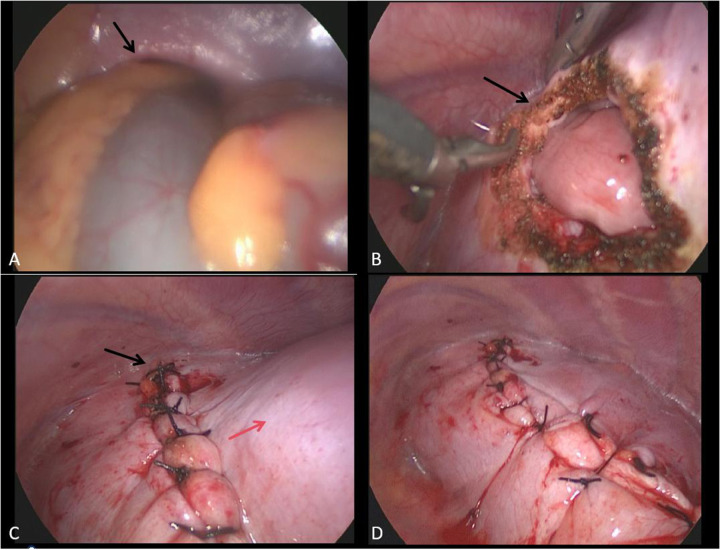
Intraoperative images. **(A)** Upon entering the thoracic cavity, bowel loops are visualized herniating through the hernia ring from the abdominal cavity; no hernia sac is present (black arrow indicates the hernia ring); **(B)** following reduction of hernia contents into the abdominal cavity, the hernia ring integrity is disrupted (black arrow indicates the disrupted hernia ring); **(C)** after closure of the hernia ring, partial elevation of the hemidiaphragm is noted (black arrow indicates the repaired hernia ring; red arrow indicates the elevated diaphragm); **(D)** final diaphragmatic configuration following repair.

### Postoperative course

Postoperative management included nasogastric decompression and parenteral nutritional support. Follow-up thoracic and abdominal CT imaging demonstrated satisfactory left lung expansion, permitting removal of the chest tube. During the first postoperative week, nasogastric drainage volumes is less than 1,200 mL daily, consisting of bilious green fluid.

On postoperative day 8, upper gastrointestinal contrast study and barium enema radiography revealed marked gastric distension with slowing of gastric peristalsis, although contrast material was observed to transit into the duodenum and small bowel. No colonic stricture was identified, and the ileocecal junction was appropriately positioned in the right lower quadrant (Figure 4).

**Figure 4:**
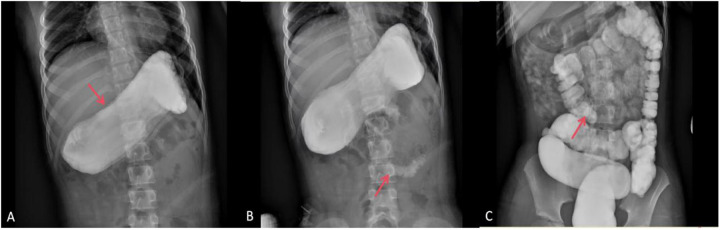
Upper gastrointestinal contrast study and barium enema radiography. **(A)** Upper gastrointestinal contrast study demonstrating marked gastric distension (arrow indicates the distended stomach); **(B)** contrast material transiting from the stomach into the small bowel, confirming absence of upper gastrointestinal obstruction (arrow indicates small bowel); **(C)** barium enema radiography showing unimpeded contrast passage through the entire colon into the small bowel, excluding lower gastrointestinal obstruction (arrow indicates the ileocecal region).

The diagnosis of postoperative gastroparesis syndrome was established. Management consisted of continuous nasogastric suction, prokinetic therapy with domperidone, and acupressure massage at the Zusanli (ST36) acupoint. Gastric drainage volumes gradually decreased to 800–1,000 mL daily. The patient demonstrated spontaneous passage of flatus and small amounts of stool during this period.

On postoperative day 15, endoscopic placement of a nasojejunal feeding tube was performed, with subsequent abdominal radiography confirming appropriate positioning of the catheter tip within the small intestine (Figure 5). Between postoperative days 16 and 21, gastric drainage progressively diminished to 200–400 mL daily.

**Figure 5:**
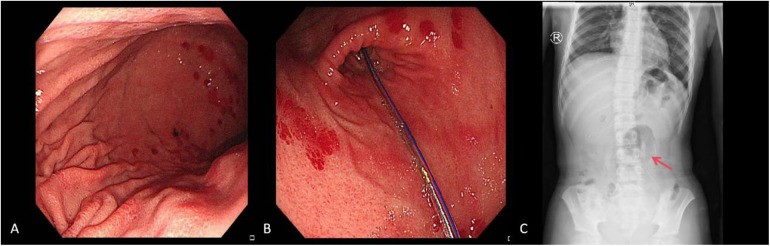
Endoscopic nasojejunal tube placement and follow-up imaging. **(A)** Endoscopic view demonstrating gastric distension; **(B)** nasojejunal tube advancement through the pylorus into the duodenum; **(C)** upright abdominal radiograph confirming catheter tip positioning within the small intestine (arrow indicates catheter tip).

On postoperative day 22, a trial of oral water intake (100 mL) was initiated with nasogastric tube clamping for 2 h followed by 30 min of drainage, monitoring output volume and character. Upon demonstrating tolerance to oral fluids, enteral intake was gradually advanced, and the nasogastric tube was clamped. The diet was progressively transitioned from clear liquids to a semi-solid consistency.

The nasogastric tube was removed on postoperative day 38, and the patient resumed a regular diet. Follow-up thoracic and upper abdominal CT imaging demonstrated normal diaphragmatic contour without evidence of fluid collection at the operative site, and marked reduction in gastric volume compared with preoperative imaging. The patient was discharged on postoperative day 44. At one-week follow-up, she had recovered well, and the nasojejunal tube was removed.

## Discussion

Thoracoscopic repair of congenital diaphragmatic hernia offers several advantages over traditional open thoracotomy, including superior visualization, reduced postoperative pain, and expedited recovery, establishing it as the predominant surgical approach for this condition (1, 2). Postoperative complications in CDH patients primarily involve the respiratory system, including pulmonary hypertension and chronic pulmonary disease (5). However, the diagnosis, management, and prognosis of gastroparesis syndrome following CDH repair remain poorly characterized in the literature.

Postoperative gastroparesis syndrome refers to functional gastric dysmotility occurring after surgery in the absence of mechanical obstruction, characterized predominantly by impaired gastric emptying. This condition most commonly follows pancreaticoduodenal procedures (3, 4). Katz et al. (6) reported a case of refractory gastroparesis in a 15-year-old patient following open repair of CDH, ultimately requiring Billroth I subtotal gastrectomy for resolution. That case established that gastroparesis can occur as a severe, medically refractory complication following CDH repair in pediatric patients. However, the patient in that report had undergone multiple prior surgical interventions—a critical confounder given that each reoperation carries independent risk for vagal or gastric injury—and the repair approach was not specifically thoracoscopic. Franken et al. (7) documented gastroparesis as a potential complication following gastrostomy placement in children requiring long-term enteral feeding. Gastroparesis has also been reported in pediatric patients following living-donor lobar lung transplantation, with potentially severe consequences if inadequately managed (8). Zhou et al. (9) described a case of PGS in a 17-year-old patient following laparoscopic choledochal cyst excision with Roux-en-Y hepaticojejunostomy, which resolved with conservative management. To our knowledge, the present case represents the first report of PGS following thoracoscopic diaphragmatic repair in a pediatric patient.

### Pathophysiology

The precise pathophysiological mechanisms underlying PGS remain incompletely elucidated but are thought to involve multiple contributing factors (10): 1. Sex-related factors: Elevated levels of estrogen and nitric oxide in females may influence gastric motility. Our patient was female. 2. Disruption of gastrointestinal 3. anatomical integrity: Alterations in digestive tract anatomy may precipitate PGS. In the present case, reduction of herniated bowel from the thoracic to the abdominal cavity may have contributed to the development of gastroparesis. 4. Psychological factors: Preoperative anxiety and heightened stress responses can induce autonomic nervous system dysfunction with sympathetic activation, resulting in impaired gastrointestinal emptying. Compared with adults, children have milder stress responses. However, the preoperative anxiety and fear of the patients might also be one of the causes. 5. Surgical denervation: Abdominal surgery may stimulate sympathetic nervous activity, suppressing gastric myoelectrical activity and delaying gastric emptying. However, given the transthoracic approach employed in this case, this mechanism is considered less likely. 6. Gastric pacemaker cell dysfunction: Surgical resection or functional impairment of interstitial cells of Cajal may compromise effective gastric pacing and motility. Although the stomach was not directly manipulated in this case, the marked gastric distension observed may have contributed to pacemaker cell dysfunction. 7. Additional factors: Significant intraoperative blood loss, postoperative metabolic derangements, analgesic administration, intra-abdominal infection, malnutrition, and psychological factors may also play contributory roles.

In the present case, we postulate that the development of PGS was multifactorial, potentially involving female sex, preoperative anxiety, gastric distension-induced pacemaker cell dysfunction, and positional changes of the bowel following reduction from the thoracic cavity. Given the paucity of reported cases, further investigation is warranted to elucidate the underlying pathophysiology.

### Diagnostic criteria

The diagnosis of PGS relies primarily on clinical manifestations including abdominal distension, vomiting, and nausea. Current pediatric diagnostic criteria are largely extrapolated from adult standards. The diagnosis may be established based on the following criteria (11):

1. Absence of underlying conditions predisposing to gastroparesis (e.g., diabetes mellitus, hypothyroidism)
2. No history of medications affecting smooth muscle contractility
3. Absence of significant metabolic derangements (e.g., fluid and electrolyte imbalances, acid-base disturbances)
4. Exclusion of mechanical obstruction by endoscopy or contrast imaging, with evidence of gastric retention
5. Nasogastric drainage exceeding 800 mL daily for more than 10 days

Our patient had no relevant past medical history, no medications known to cause gastroparesis, and no electrolyte abnormalities. Postoperative contrast imaging demonstrated significant gastric retention with delayed emptying, and upper endoscopy excluded mechanical obstruction. Nasogastric drainage consistently exceeded 800 mL daily during the first two postoperative weeks, confirming the diagnosis of PGS following thoracoscopic diaphragmatic repair.

### Management

Postoperative gastroparesis syndrome represents a functional, non-obstructive disorder. Current therapeutic approaches in pediatric patients focus on treating the underlying condition, optimizing nutritional status, preventing electrolyte disturbances, and alleviating symptoms (12).

Patients with mild PGS may benefit from dietary modifications, including frequent small meals with low-fat, low-fiber content. Nasojejunal tube feeding may be instituted when necessary to provide enteral nutrition (13). Prokinetic agents including metoclopramide, domperidone, cisapride, prucalopride, and macrolide antibiotics have demonstrated efficacy in promoting gastrointestinal recovery in pediatric PGS (14, 15). Acupuncture at the Zusanli (ST36) point has been reported to regulate gastric myoelectrical rhythm and promote gastric emptying (16). Endoscopic pyloric dilation and botulinum toxin injection have also shown effectiveness in pediatric gastroparesis (17). Surgical interventions such as gastrostomy or jejunostomy may be considered in patients refractory to conservative management (18).

Current literature indicates that gastroparesis is a known but not yet fully clarified complication among survivors of congenital diaphragmatic hernias, and surgical procedures within the thoracic cavity may cause delayed gastric emptying. However, there have been no previous reports describing the occurrence of gastroparesis in pediatric patients after a single, uncomplicated thoracoscopic repair of congenital diaphragmatic hernia. This case fills this specific gap and provides the first detailed clinical description of postoperative gastroparesis in this specific clinical setting.

In the present case, the patient was managed with continuous nasogastric decompression, nasojejunal tube placement for enteral nutrition, parenteral nutritional support to maintain metabolic homeostasis, oral domperidone for prokinetic effect, and Zusanli acupoint massage. The patient achieved complete recovery with conservative management and was discharged in good condition.

However, the limitation of this study is that it is a case study, making it impossible to determine the risk factors for PGS in children. Additionally, since children with PGS are relatively few, there are also fewer related literature studies. The diagnosis of vagus nerve injury is still at a speculative stage; in the clinical setting, vagus nerve injury cannot be directly confirmed, and the intraoperative integrity detection technology of the vagus nerve used in certain fore-stomach surgeries has not been carried out. It is impossible to clearly distinguish the specific influence of pre-existing anatomical factors and intraoperative events on the formation of gastric emptying disorders. Moreover, due to the retrospective nature of this report, detailed intraoperative records of the visualization process of the vagus nerve and the precise range of the esophageal surrounding dissection cannot be obtained. In this study, the treatment methods for patient were based on those for adults. For children, whether there are more effective treatment methods requires further research.

## Conclusion

Postoperative gastroparesis syndrome is a functional disorder that rarely complicates thoracoscopic diaphragmatic hernia repair. Prompt recognition and diagnosis are essential when this complication occurs. Comprehensive non-operative management, including enteral and parenteral nutritional support, gastric decompression, and maintenance of metabolic stability, facilitates patient recovery. Given the limited literature on late-presenting CDH in older children and the paucity of data regarding post-CDH gastroparesis, long-term follow-up and surveillance are recommended to optimize outcomes in this patient population.

## Data Availability

The original contributions presented in the study are included in the article/Supplementary Material, further inquiries can be directed to the corresponding author.
