# Supplementary figures and images for: Gastroparesis syndrome following thoracoscopic diaphragmatic hernia repair in a pediatric patient: a case report and literature review
Source: Front Pediatr. 2026 May 29;14:1813576. doi: 10.3389/fped.2026.1813576 (PMC13260072; doi:10.3389/fped.2026.1813576)

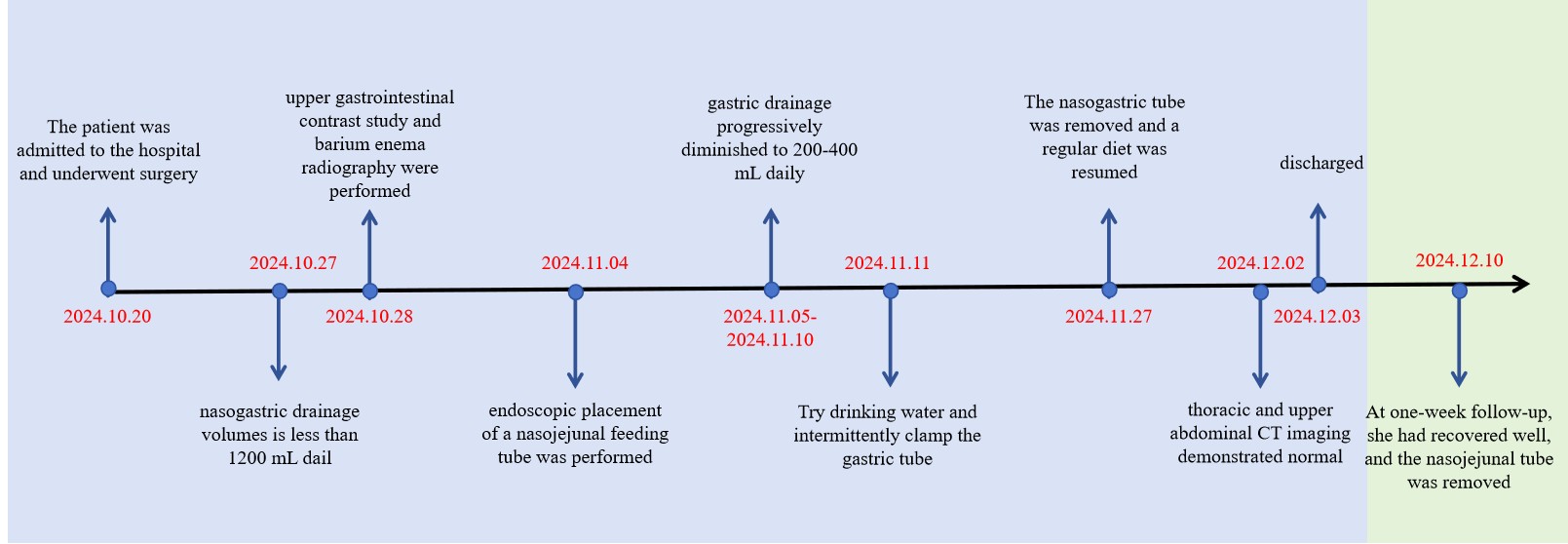

Supplement: Supplementary file 1 [file Image1.jpg]
